# Ion-Driven Electrochemical Random-Access Memory-Based Synaptic Devices for Neuromorphic Computing Systems: A Mini-Review

**DOI:** 10.3390/mi13030453

**Published:** 2022-03-17

**Authors:** Heebum Kang, Jongseon Seo, Hyejin Kim, Hyun Wook Kim, Eun Ryeong Hong, Nayeon Kim, Daeseok Lee, Jiyong Woo

**Affiliations:** 1 School of Electronic and Electrical Engineering, Kyungpook National University, Daegu 41566, Korea; hbkang@knu.ac.kr (H.K.); hw.kim@knu.ac.kr (H.W.K.); er.hong@knu.ac.kr (E.R.H.); 2 Department of Electronic Materials Engineering, Kwangwoon University, Seoul 01897, Korea; seojs@kw.ac.kr (J.S.); hyejinkim@kw.ac.kr (H.K.); 3 School of Electronics Engineering, Kyungpook National University, Daegu 41566, Korea; nayeon.kim@knu.ac.kr

**Keywords:** electrochemical RAM, redox transistor, neuromorphic computing, synaptic devices

## Abstract

To enhance the computing efficiency in a neuromorphic architecture, it is important to develop suitable memory devices that can emulate the role of biological synapses. More specifically, not only are multiple conductance states needed to be achieved in the memory but each state is also analogously adjusted by consecutive identical pulses. Recently, electrochemical random-access memory (ECRAM) has been dedicatedly designed to realize the desired synaptic characteristics. Electric-field-driven ion motion through various electrolytes enables the conductance of the ECRAM to be analogously modulated, resulting in a linear and symmetric response. Therefore, the aim of this study is to review recent advances in ECRAM technology from the material and device engineering perspectives. Since controllable mobile ions play an important role in achieving synaptic behavior, the prospect and challenges of ECRAM devices classified according to mobile ion species are discussed.

## 1. Introduction

**Figure 1:**
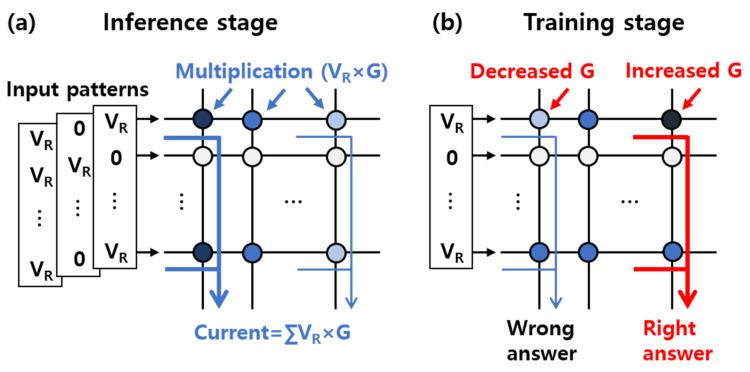
Neuromorphic systems based on (**a**) the inference and (**b**) training stages. During the inference step, the input voltage vector–G matrix multiplication occurs at each cross, and the currents through the columns are compared. Contrary to expectations, the current in the other column may become the highest, which leads to an incorrect answer. The systems are trained to derive the correct values by properly adjusting the G level of the synaptic element.

As electronic devices are widely distributed in our society, information is shared through connected devices, generating an explosive amount of data. This promotes the rapid development of memory technologies and demands faster and higher memory density for data centers and the Internet of Things. Furthermore, artificial intelligence recognizing regular patterns from massive data has been introduced to autonomous vehicles. Specifically, not only are high-density memory-implemented chips needed to drive the cars by continuously acquiring data but the chips should also execute the stored data as quickly as possible for real-time object detection. The state-of-the-art memory capacity has been dramatically increased to greater than terabyte because of 3D NAND FLASH, but the latency bottleneck occurs during the numerous data transfers between the processor and memory units. To alleviate this serial execution based on frequent data movement in the von Neumann computing architecture, neural network algorithms, which are inspired by a human brain structure, have been suggested [1]. In a biological brain, a neuron generates a signal and addresses it to the next neurons through synapses. Since the multiple synapses are connected to the neuron, the signal can be simultaneously distributed in a parallel fashion, thereby consuming extremely low power. This neural network can be artificially built by constructing a crosspoint (or bar) array architecture, where an electronic memory device serving as the synapse is positioned between two perpendicular row and column lines [2]. As the input voltage as the signal comes to the crosspoint array through the row line, the current becomes the output due to multiplication between the voltage and conductance (G) stored at the synaptic element. The output current at the end of the column line can be low or high depending on what input combinations are applied in the row at a given G map assigned to the synaptic array. In other words, input patterns, which can be decomposed into an input voltage vector, can be classified by figuring out the largest output column current, which is the inference stage based on the propagation algorithm, as shown in Figure 1a. When the G level of the synaptic element is divided into multiple values, the output current can be distinguished more sensitively, ensuring high classification accuracy.

For accelerating this inference, various emerging memories, such as magnetic memory, phase-change memory (PCM), and resistive-switching memory (RRAM) have been suggested for the synaptic element [2,3,4]. The magnetic memory technology primarily based on spin-transfer torque has been mass-produced for embedded memory products [5], but the binary resistance state has become a drawback. The G matrix mapped with binary values may enable the designed systems to recognize relatively simple patterns, such as handwritten images, rather than universal applications. However, multiple resistance states can be achieved in PCM based on multicomponent chalcogenide by elaborately controlling the volume fractions of the amorphous and crystalline phases [6]. However, since the switching dynamics are basically driven by thermal Joule heating induced by the operating current, the high power consumption needs to be reduced by adding dopants, such as nitrogen [7], making the materials even more complicated. In this regard, RRAM that exploits electrically controllable growth and dissolution of conductive filaments with a low-operating-current regime can be an alternative [8,9]. More than 32 levels in RRAM achieved by fine-tuning the current or voltage have, thus, been reported.

**Figure 2:**
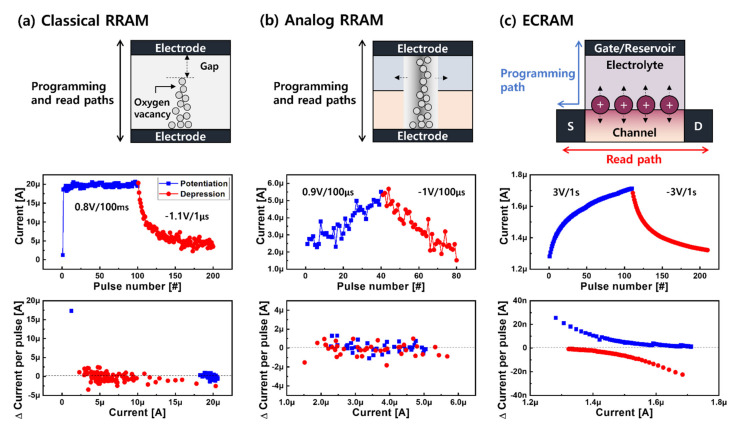
Synaptic responses of (**a**) classical RRAM [12], (**b**) analog RRAM [12], and (**c**) ECRAM [19]. The first row shows the schematic diagram of each device. The second row shows the synaptic responses of the devices as a function of the identical pulse technique. The change in current in every pulse step is shown in the third row.

As shown in Figure 1b, contrary to expectations, the output current in the wrong column becomes the largest, inducing classification error. The system needs to newly update the preexisting G level in order to derive the correct answer on the basis of the backpropagation algorithm; this is called the training phase. For achieving higher training accuracy, how the analog resistance states are changed is important rather than the number of states [10,11]. Considering the problem of circuitries, changing G is preferred, which is performed by an identical pulse technique, which only varies the number of pulses at fixed amplitude and width. According to the literature, the behavior of the update curve of the current state is convex upward (or downward), which means that the extent of the change in G for every signal becomes smaller (or higher), as shown in Figure 2. With classical RRAM based on filaments, a highly nonlinear update response has been mainly observed [12], which can be classified into two distinct scenarios: abrupt increase and being stuck at saturation (Figure 2a). This is because clustering of oxygen vacancies instantly makes a path due to the highly concentrated electric field at the small gap between the electrode and partially grown filament [13]. When the RRAM showed metallic properties through the fully formed filament, the applied field became uniformly distributed across the whole stack instead of the local gap, making it difficult to effectively change the size of the filament. Unlike potentiation corresponding to the increase in current (or G), gradual depression, which was equivalent to the decrease in current, was observed because the filament was radially dissolved by the thermal energy, as well as the opposite field. This asymmetric identical pulse response in the classical RRAM is one of the causes of the degradation of the pattern recognition accuracy. It can be alleviated by introducing analog RRAM, where the size of the filament evolves in the lateral direction. As shown in Figure 2b, nearly linear potentiation and depression characteristics are mainly observed in the bilayer structure, such as HfO_x_/AlO_x_ [12] and TaO_x_/HfO_x_ [14]. Although the same amount of current was changed in every pulse step, the intrinsic filamentary nature based on stochastic ion motion caused variability. Additionally, the updated states were identified by addressing a smaller read pulse through the same location as when it was programmed, worsening the state’s nonuniformity.

Therefore, electrochemical random-access memory (ECRAM) [15,16] has been designed using a three-terminal structure to intentionally decouple the programming and read paths, as shown in Figure 2c. ECRAM operation seems to be similar to a conventional transistor, where the gate controls the channel G, but a solid electrolyte that facilitates ion movements driven by an electric field is used instead of a gate dielectric. Thus, it allows the changed G level to be maintained even without removing the voltage, resulting in nonvolatility. To modulate channel G, the voltage was applied between the gate and the source. The channel G level was read laterally by the voltage application between the source and drain. The mobile ions, which are typically included inside the electrolyte, can be moved back and forth toward the channel in the vertical direction. It has not yet been clearly elucidated how the moved mobile ions are exactly involved in converting the channel G. Generally, it has been described as two scenarios. On the one hand, electrons can easily flow through the accumulated mobile ions near the channel, creating a lateral conductive path [17]. On the other hand, ions serving as dopants that reach the channel chemically react with channel atoms [15]. The valence of the channel atom can be, thus, smaller or higher, resulting in an electrically conductive or insulative path. To date, various mobile ions have been explored. Then, an ECRAM stack that can effectively control the specifically selected ions has been designed. The ECRAM first used Li ions from a thin-film rechargeable battery. Li-ion-incorporating channel and electrolyte materials, such as lithium phosphorus oxynitride (LiPON), were used. However, considering CMOS compatibility, new mobile ions, such as oxygen ions (or vacancies) [18] and Cu ions [19], have been studied. Considering recent advancements of the ECRAM, in this study, the progress and prospect of each type of ECRAM classified according to the use of mobile ions were discussed.

## 2. Results and Discussion

### 2.1. Li-Ion-Based Electrochemical Random-Access Memory

**Figure 3:**
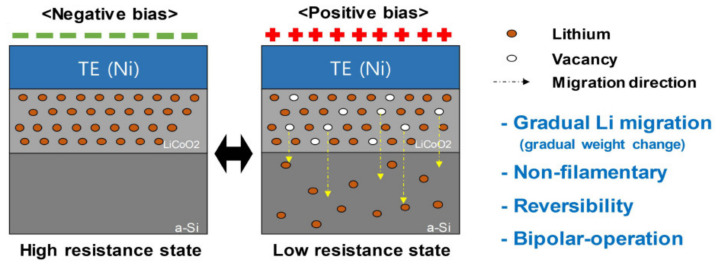
Simple illustration of the operation mechanism in the 2T Li-ECRAM [20]. Copyright IEEE, 2019.

Li-ion-based ECRAM was proposed implementing the charge/discharge mechanism of a Li-ion battery [15,16]. During the charge and discharge processes, the redox reaction of the Li ion occurs at both the anode and the cathode, which can lead to resistance changes of the anode or cathode. However, multiterminal Li-ion-based ECRAM with more than three terminals can increase circuit complexity. Thus, in the device structure, a two-terminal Li-ion-based ECRAM (2T Li-ECRAM) was proposed for high integration density [20,21]. Even in the two-terminal structure, G can be controlled by a migration of the Li ions, as shown in Figure 3. Under applied external positive bias, the Li ions are oxidized in the LiCoO_2_ layer (cathode) and move into an amorphous silicon layer (anode) and vice versa. It is a totally reversible process, and the concentration of the Li ion is gradually changed in the LiCoO_2_ layer during the process. When the loss of Li ions increases, the LiCoO_2_ layer can be expressed as Li_1−x_CoO_2_, where “x” implies a vacancy formation which can increase the G of the Li_1-x_CoO_2_ layer. In other words, under external positive bias, the G level of the Li_1−x_CoO_2_ layer increases because of the increased loss of Li ions in the LiCoO_2_ layer, which leads to increased total G of the 2T Li-ECRAM.

**Figure 4:**
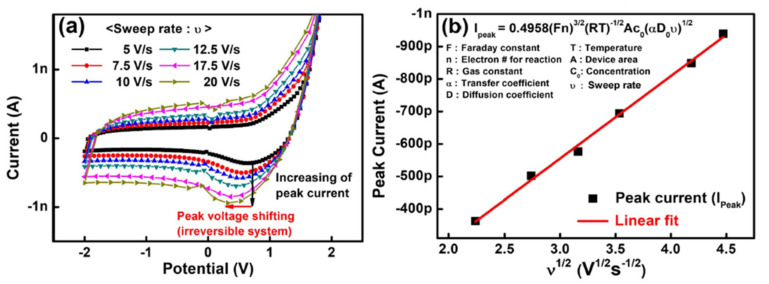
(**a**) Cyclic voltammetry with various sweep rates of the Li-ion-based two-terminal synaptic device. (**b**) Peak currents of the cyclic voltammetry fitted by the Randles–Sevcik equation [20]. Copyright IEEE, 2019.

The Li-ion migration can be confirmed by measuring cyclic voltammetry, as shown in Figure 4. The 2T Li-ECRAM exhibited cyclic voltammetry with various sweep rates, which can be explained as the redox reaction of the ions. To confirm the faradaic current derived from the ion migration, the peak currents of the cyclic voltammetry were fitted by the Randles–Sevcik equation.

(1) $$I_{peak}=0.4958{\left(Fn\right)}^{\frac{3}{2}}{\left(RT\right)}^{-\frac{1}{2}}Ac_{0}{\left(\alpha D_{0}\nu \right)}^{\frac{1}{2}},$$

where *I*_peak_ is the peak current, *F* is the Faraday constant, *n* is the number of electrons transferred in the redox event, *R* is the gas constant, *T* is the temperature, *A* is the area, *C*_0_ is the ion concentration, *α* is the transfer coefficient, *D*_0_ is the diffusion coefficient, and *ν* is the scan rate. The peak current was linearly proportional to the sweep rate, indicating that it was an ionic current.

**Figure 5:**
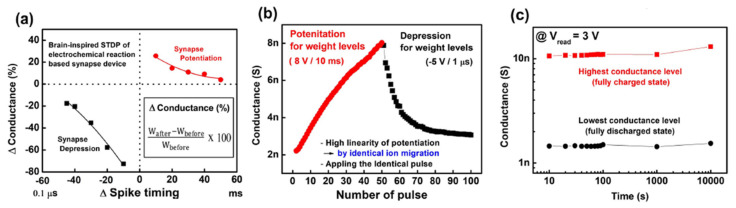
(**a**) Spike-timing-dependent plasticity of the Li-ion-based two-terminal synaptic device. (**b**) Potentiation and depression characteristics based on restricted Li-ion migration. (**c**) Long-term retention at the highest and lowest G levels [20]. Copyright IEEE, 2019.

From the controlled Li-ion migration, synaptic characteristics can be obtained, as shown in Figure 5. Spike-timing-dependent plasticity that is dependent on the timing difference between spikes from pre- and post-neurons is one of the representative synaptic characteristics. The timing difference can result in different strengths of synapse connections (synaptic weight). In other words, at the synapse, coherent spikes increase the synaptic weight more than incoherent spikes. Figure 5b shows the potentiation and depression characteristics under sequentially applied 50 identical potentiation pulses and 50 identical depression pulses. The 2T Li-ECRAM exhibited gradual G levels, which can be considered as the synaptic weight in the hardware implementation. Additionally, the G levels were maintained for 10,000 s, suggesting nonvolatile G levels.

However, during the depression, the 2T Li-ECRAM exhibited a nonlinear G decrease, which can degrade the recognition accuracy of the hardware-implemented neuromorphic algorithm. Nonlinear depression can result from the self-discharge behavior of the ECRAM. When the Li ion is transferred from the cathode to the anode, an electromotive force is formed in the device cell, which forces the Li ion to move back to the cathode. Consequently, more abrupt G changes were observed during depression [20].

**Figure 6:**
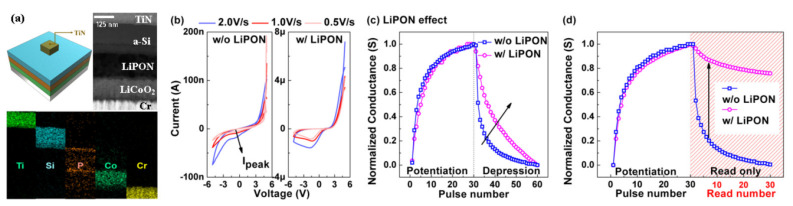
(**a**) Cross-sectional transmission electron microscopy image of the 2T Li-ECRAM including the LiPON electrolyte. (**b**) Comparison of cyclic voltammetry with and without the electrolyte. (**c**) Improved depression linearity by inserting the electrolyte. (**d**) Comparison of retention at the maximum G level. The improved results come from the self-discharge behavior prevented by the electrolyte [21]. Copyright IEEE, 2019.

**Figure 7:**
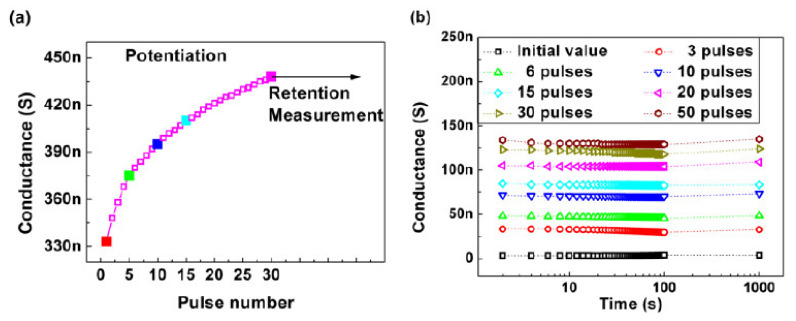
(**a**) Selected multilevel G for retention measurement. (**b**) Stable multilevel G retention from the electrolyte [21]. Copyright IEEE, 2019.

To prevent the self-discharge behavior of the 2T Li-ECRAM, a solid-state electrolyte was adopted between the cathode and anode [21]. Figure 6a shows a cross-sectional transmission electron microscopy image of the device cell. The LiPON layer was inserted between the LiCoO_2_ and a-Si layers as an electrolyte. By inserting the electrolyte, more stable oxidations were observed under cyclic voltammetry, as shown in Figure 6b. Moreover, the abruptly changed depression was improved (Figure 6c). These results come from the limited self-discharge behavior; the maximum G level was maintained for a longer time by inserting the electrolyte, as shown in Figure 6d. At various G levels, the self-discharge behavior was prevented, which led to stable G retention, as shown in Figure 7.

The Li-ion-based electrochemical synaptic devices exhibit obviously improved synaptic characteristics such as potentiation/depression linearity, cycle endurance, and multistate retention. Even though the Li-ion-based electrochemical synaptic devices have various advantages, additional requirements should be satisfied for the hardware implementation of the neuromorphic algorithm. Because they are operated by Li-ion migration, Li-ion-based electrochemical synaptic devices have a relatively slow switching speed from microseconds to milliseconds. For lower energy consumption of the hardware implementation, a faster switching speed needs to be realized. Additionally, for high integration density and mass production process, CMOS-compatible materials are required. The materials consisting of Li ions are not proper for a typical CMOS process. Thus, more CMOS process-compatible materials and device structures are necessary. Lastly, the dynamic range, which is the difference between the maximum and minimum G levels, can affect the accuracy of the hardware-implemented neuromorphic algorithm. Therefore, the dynamic range of the Li-ion-based electrochemical synaptic devices needs to be enlarged.

### 2.2. Oxygen-Ion-Based Electrochemical Random-Access Memory

In 2013, the preliminary ion-driven ECRAM was demonstrated using a combination of an ionic liquid and a perovskite SmNiO_3_ channel material [22]. In the material system, the channel G level could be modulated by electrochemical reactions at the ionic liquid/SmNiO_3_ channel interface as follows:

O_O_^x^ ↔ Vo^2+^ + 2e^−^ + ½O_2_. (2)

Ni^3+^ + e^−^ ↔ Ni^2+^. (3)

When oxidation occurred in the oxide under voltage application, the gate attracted the oxygen ion with negative polarity. The electrons created by the vacancy as oxygen was released reduce Ni^3+^ to Ni^2+^ for stabilization, transforming the metallic channel state. As a result, successive gate pulses with +2.5/−2.5 V and a width of 10 ms linearly controlled the channel G level with a range of 1,000% (Table 1).

However, considering the integration perspective, the use of a liquid electrolyte is vulnerable to external ambient conditions such as humidity. Thus, a metal-oxide-based ECRAM was fabricated with a HfO_2_/WO_3_ stack [18]. The G level with few tens of range was tuned by 20 pulses with a width of 0.5 s. Interestingly, the synaptic characteristics were shown even after annealing at a high temperature of 400 °C. The ECRAM continued to be updated after ~2 × 10^7^ pulses without degradation. The G response was a function of pulse width, which was varied from 10 µs to 10 ns. Furthermore, reliable cycling behavior was achieved by more than 10^7^ pulses.

Pr_1−x_Ca_x_MnO_3_ (PCMO) is known as an ionic conductor that promotes the migration of oxygen ions [23]. Usually, the resistive-switching behavior based on the valence change of the Mn oxidation states between Mn^3+^ and Mn^4+^, corresponding to insulative and conductive states, respectively, has been understood in a two-terminal structure, where PCMO is sandwiched between electrodes [24,25]. This relatively clarified area switching phenomenon was exploited in a three-terminal structure [26]. Polycrystalline PCMO annealed at 600 °C served as the channel layer. Since PCMO was regarded as a p-type oxide semiconductor, the oxygen-deficient state showed low conductivity. Lee et al. [26] first investigated the impact of annealing conditions on the G level of PCMO to identify the origin of analog switching. When forming gas annealing was performed, the G level of about 250 nS was lowered with respect to the temperature. The lowered G level was reversibly recovered by supplying oxygen, indicating that the oxygen ion movement plays an important role. When a HfO_x_ electrolyte with a GdO_x_ reservoir was inserted on top of PCMO, the channel G level could be modulated by electrical pulses. The positive gate voltage moved the oxygen anions back to the reservoir, decreasing G. On the contrary, the negative voltage drove the ions toward the channel, increasing G.

On the basis of the switching mechanism, the impact of electrolytes was further analyzed. Specifically, the electrolyte density, which was involved in the extent of ion migration, was adjusted [27]. During the HfO_x_ electrolyte deposition, the working pressure was varied in the range of 3–15 mTorr. The results showed that a high-quality HfO_x_ film with less porosity was obtained at lower working pressure. Note that the porosity of the electrolyte was inversely proportional to the synaptic behavior. Gate pulses higher than 4 V only activated the switching behavior in the PCMO synaptic device with the HfO_x_ film deposited at 3 mTorr working pressure condition. The driving gate voltage was reduced using an HfO_x_ layer deposited at higher working pressure. This means that the oxygen ion migration was preferred through the boundary, such that the ion could be easily moved through the defective HfO_x_ layer.

Channel quality was investigated in addition to the effect of the electrolyte [28]. With the given YSZ electrolyte acting as a good ion conductor, two binary oxides, TiO_x_ and WO_x_, were compared. When the oxygen-rich WO_3_ channel was used, the G level began to be easily saturated after a few gate pulses, constraining a small G window. Furthermore, the oxygen-deficient WO_x_ channel layer promoted oxygen ion migration by the lowered vacancy formation energy and migration barrier, leading to a continuous change in G. The impact of the stoichiometry of the channel material was further verified using another binary oxide, i.e., a TiO_2_ layer. The oxygen-deficient channel layer induced more active redox reactions under voltage application, accelerating the ion diffusivity. Note that the achieved results were based on polycrystalline channel oxides; hence, the amorphous phase could provide a different electrical response.

**Table 1:** Benchmark table for oxygen-ion-driven electrochemical random-access memories.

Device stack	Electrolyte	Ionic Liquid	HfO_2_	HfO_2_	HfO_1.74_	HfO_x_	YSZ
	Channel	SmNiO_3_	WO_3_	WO_X_	PCMO	PCMO	TiO_x_
Mobile ion		Oxygen ion	Oxygen ion	Oxygen ion	Oxygen ion	Oxygen ion	Oxygen ion
Conductance range		1.1	20	~6	~1.75	~2.25	7
Driving conditions	Potentiation	−2.5 V/10 ms	+1 nA/0.5 s	+4 V/1 s	−3.75 V/1 s	−3.5 V/100 ms	+4 V/500 ms
	Depression	+2.5 V/10 ms	−1 nA/0.5 s	−3 V/1 s	3V/1 s	2.5 V/100 ms	−3.5 V/500 ms
Reference		[22]	[18]	[29]	[26]	[27]	[28]

### 2.3. Proton-Based Electrochemical Random-Access Memory

To clarify the role of H in the synaptic device, hydrogen-doped SiO_x_ electrolytes with different hydrogen concentrations were used above the WO_x_ channel [30]. Hydrogen was incorporated into the electrolyte by sputtering with a SiO_2_ target under Ar and forming gas. The hydrogen concentration could be, thus, changed by fine-tuning the ratio of the two gases. In the capacitance–voltage measurement with a frequency of 1 kHz, hydrogen-doped SiO_2_ only exhibited a hysteresis loop, implying that hydrogen was driven by the applied field.

Since hydrogen serves as the mobile source, organic materials have been mainly used for the ECRAM specifically aimed at wearable and flexible applications. The positive voltage to poly(3,4-ethylenedioxythiophene):polystyrene sulfonate (PEDOT:PSS) acting as the gate electrode pushed hydrogen toward PEDOT:PSS with a polyethylenimine (PEI) channel [31]. This caused the reduction of PEDOT, decreasing the conductivity of the PEDOT:PSS/PEI channel. The multiple G states were linearly modulated by more than 500 pulses with a low driving voltage of hundreds of millivolts. A switching energy of about 10 pJ was experimentally achieved and was further expected to be projected to 35 aJ at a sub-micrometer scaled-down device area of 0.3 µm × 0.3 µm.

The organic ECRAM can be composed of a semiconducting polymer, such as PEDOT:PSS separated by Nafion, which is a solid electrolyte [32]. Electron injection and extraction due to hydrogen migration induced a redox reaction at the PEDOT:PSS channel, tuning G. Fifty G states could be adjusted by a low voltage of ±650 mV, and a larger voltage of ±1 V allowed a twofold higher dynamic range.

Although these devices demonstrated low-voltage operation (Table 2), CMOS-compatible fabrication processing and reliability need to be taken into account. In this regard, Yao et al. [33] reported hydrogen intercalation exploited in inorganic material systems with a Nafion-117 electrolyte and a WO_3_ channel. Interestingly, palladium hydride (PdH_x_) was used for the reservoir of hydrogen. Hydrogen was introduced by exposing the deposited Pd layer to forming gas ambient. The gate pulse started to oxidize PdH_x_, and the released protons were transferred to the channel through the Nafion electrolyte. The injected hydrogen to the WO_3_ lattice created defects by bonding with the oxygen ion, increasing the conductivity in the channel.

### 2.4. Cu-Ion-Based Electrochemical Random-Access Memory

Cu ions as a new mobile ion source can be supplied from the interconnect in the back-end-of-line process. Nonvolatile memory behavior based on Cu ion motion has been intensively studied for a two-terminal configuration, called conductive-bridge RAM (CBRAM) [35,36]. Because of its fast operating speed, the CBRAM is expected to be a key element for storage-class memory [36] or reconfigurable applications [37]. Additionally, an atomic transistor concept was proposed for logical functions [17]. In the three-terminal transistor structure, the Cu ions are supplied from the Cu gate electrode; thus, the ions passing through the Ta_2_O_5_ solid electrolyte are accumulated at the channel (Table 3). The nucleation of Cu electrically bridges the channel, which leads to an instantly high current on the order of about 10^4^ flowing between the source and drain electrodes, thereby turning on the switch.

Studies utilizing Cu ions have regained attention due to the demand for manufacturing compatibility of the ECRAM. To make the Cu ions move uniformly across the entire area, unlike CBRAM operation, highly conductive solid-state electrolytes of the Cu–Rb–I–Cl system were introduced for the analog synapse [38]. The lithium-free solid-state Cu-ion-actuated ECRAM synapse exhibited analog switching by a small programming pulse amplitude of 100 mV, which might have been the result of the faster ion mobility and ionic conductivity of the electrolyte.

**Figure 8:**
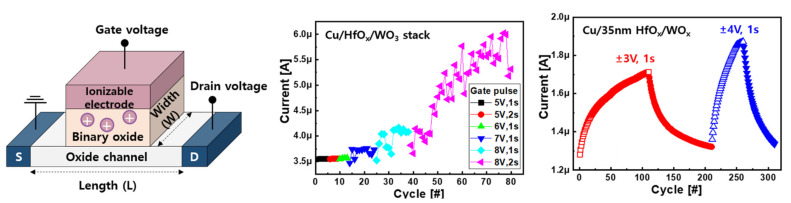
Cu-ion-driven three-terminal structure with Cu/HfO_x_/WO_x_. The channel current begins to increase when a gate voltage above a certain threshold is applied. The uniformly increased channel current can be achieved by the stoichiometry of the channel material. The magnitude of the current increase is adjusted by the gate voltage amplitude [19]. Copyright AIP, 2021.

**Figure 9:**
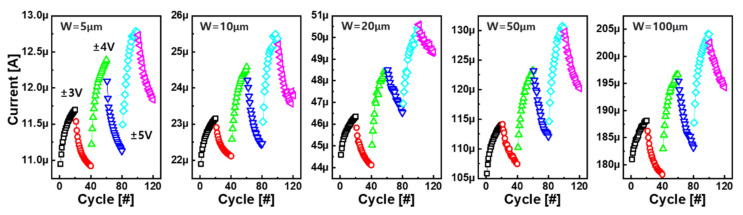
Area-scalable synaptic response. As the width of the channel is expanded, the channel current becomes proportionally larger [19]. Copyright AIP, 2021.

Recently, analog switching was demonstrated in a fully CMOS-compatible stack with a Cu gate electrode/HfO_x_ electrolyte/WO_x_ channel structure [19], as shown in Figure 8. It is believed that the valence change of the W atom at the channel with respect to Cu ions as a dopant was involved. The channel current began to respond when the gate voltage was above a critical criterion of 6 V, indicating field-induced ion migration. Although the current was increased by the gate, fluctuation of the channel current was observed, indicating poor gate controllability on the channel. The nonuniform current response could be improved by lowering the channel resistance to apply most of the gate voltage to the electrolyte. This was experimentally achieved by depositing a nonstoichiometric WO_x_ channel layer by sputtering a single WO target with only Ar plasma instead of reactive sputtering to the W metal target with oxygen and argon gases. As a result, the channel current of the Cu/HfO_x_/WO_x_ ECRAM continued to increase (or decrease) analogously by the number of positive (or negative) gate pulses. Since ion movement played a vital role, the degree of the current change was steadily enlarged by the larger gate voltage amplitude at a given pulse width. The area-scalable synaptic response is shown in Figure 9. The channel current became smaller when the channel width was shortened from 100 to 5 µm at the given length of 10 µm. The read current at 0.5 V decreased linearly, implying area switching.

## 3. Conclusions

In this mini-review, ECRAM-based synaptic devices for neuromorphic computing applications were discussed. Inspired by Li-ion intercalation in a secondary ion battery, various studies have attempted to develop suitable Li-incorporated electrolyte materials such as LiTiO_x_ [39] and LiPOSe_x_ [40]. Furthermore, since specific oxidation or reduction is thermodynamically preferred, an asymmetric potentiation and depression behavior was observed. To mitigate this challenge, an ion-controllable thin Al_2_O_3_ barrier was introduced [41]. Recently, to overcome the drawback of the three-terminal structure at the expense of analog synaptic properties, uniform Li-ion motion was realized in a two-terminal ECRAM. In the case of using hydrogen ions, which are mainly contained in polymers, the organic ECRAMs were operated at very low voltage. All organic-based ECRAMs are expected to be used for specific applications, where solution and roll-to-roll processing are required. Considering CMOS compatibility, oxygen- and Cu-ion-based ECRAMs can be integrated into a large-scale array. By analyzing the material aspects of the electrolyte and channel layer, a deep understanding of the factors on the linearity and symmetry of the synaptic properties has been gained. Since the area-scalable switching behavior has been observed, the switching speed can be expected to be faster in the aggressively scaled devices, which needs to be verified. By introducing an optimized structure, the speed and dynamic range of the conductance can be also improved. A structure that efficiently generates heat and confines it in the ECRAM can promotes ion movement, resulting in faster switching speed. To date, physics-based modeling [42] has rarely been studied except for the equivalent circuit model [43]. A model that fits well with the experimental results will not only allow the design exploration of the synaptic devices for further improvement but also extrapolate the reliability perspective of ECRAMs. ECRAM has been proposed to accelerate training due to the linear G update, known as one of the key factors for achieving high recognition accuracy, but it may be difficult to satisfy all reliability criteria. Depending on the systems for edge or server computing, inference can mainly be performed, while training can be conducted infrequently. This means that the stability of multiple G states may be more important than cycling endurance. When the ECRAM is designed for a specific system purpose, hardware demonstration of the neuromorphic chip is expected to be accelerated.

## Figures and Tables

**Table 2:** Benchmark table for hydrogen-ion-driven electrochemical random-access memory.

Device stack	Electrolyte	Ionic Liquid	PEDOT:PSS	Nafion	SiO_x_	Nafion
	Channel	MoO_3_	PEDOT:PSS/PEI	PEDOT:PSS	WO_2.7_	WO_3_
Mobile ion		Hydrogen	Hydrogen	Hydrogen	Hydrogen	Hydrogen
Conductance range		~1.35	~1.5	~2	~6	~4
Driving conditions	Potentiation	+2.5 V/1 ms	−100 mV	−1.1 V/50 ms	+3 V/1 s with -1 V/0.5 s	+0.25 V/5 ms
	Depression	−1.8 V/1 ms	+100 mV	+1 V/50 ms	−2.5 V/1s with +1 V/0.5 s	−0.25 V/5 ms
Reference		[34]	[31]	[32]	[28]	[33]

**Table 3:** Benchmark table for Cu-ion-driven electrochemical random-access memory.

Device Stack	Cu/Ta_2_O_5_	Cu/Cu_6_Rb_41_Cl_13_/TiN (or TaN)	Cu/HfO_x_/WO_x_
Volatility	Nonvolatile	Nonvolatile	Nonvolatile
Resistance state	Binary	Analog	Analog
Switching mechanism	Forming and dissolution of Cu nucleus	Plating and deplating of Cu	Valence change
Applications	Logic	Analog synapse	Analog synapse
Reference	[17]	[38]	[19]

## Data Availability

Not applicable.
